# Radiological assessment of effectiveness of soluble RAGE in attenuating Angiotensin II-induced LVH mouse model using *in vivo* 9.4T MRI

**DOI:** 10.1038/s41598-019-44933-6

**Published:** 2019-06-11

**Authors:** Dan Heo, Soyeon Lim, Jiye Lee, Myung Eun Lee, Soyoung Cho, Jisu Jeong, Miran Seo, Sungha Park, Jaemoon Yang

**Affiliations:** 1 Department of Radiology, Yonsei University College of Medicine, Seoul, Republic of Korea; 2 Institute for Bio-Medical Convergence, College of Medicine, Catholic Kwandong University, Gangneung, Gangwon-do Republic of Korea; 3 Integrative Research Center for Cerebrovascular and Cardiovascular diseases, Yonsei University College of Medicine, Seoul, Republic of Korea; 4 Graduate Program in Science for Aging & Yonsei Research Institute of Aging Science, Yonsei University, Seoul, Republic of Korea; 5 Cardiovascular Research Institute, Yonsei University College of Medicine, Seoul, Republic of Korea; 6 Division of Cardiology, Yonsei University College of Medicine, Seoul, Republic of Korea; 7 Brain Korea 21 Project for Medical Science, Yonsei University, Seoul, Republic of Korea; 8 Systems Molecular Radiology, Yonsei University, Seoul, Republic of Korea

**Keywords:** Cardiac hypertrophy, Experimental models of disease

## Abstract

We investigated the effectiveness of soluble Receptor for Advanced Glycation Endproducts (sRAGE) in attenuating angiotensin II (AngII)-induced left ventricular hypertrophy (LVH) using *in vivo* 9.4T cine-magnetic resonance imaging (CINE-MRI). Mice were divided into four groups: AngII (n = 9), saline (n = 10), sRAGE (n = 10), and AngII + sRAGE (n = 10). CINE-MRI was performed in each group after administration of the AngII or sRAGE, and CINE-MR images were analyzed to obtain parameters indicating cardiac anatomical and functional changes including end-diastolic and end-systolic blood volume, end-diastolic and end-systolic myocardial volume, ejection fraction, end-diastolic and end-systolic myocardial mass, and LV wall thickness. LVH observed in AngII group was significantly attenuated by sRAGE. These trends were also observed in histological analysis, demonstrating that cardiac function tracking using *in vivo* and real-time 9.4T MR imaging provides valuable information about the cardiac remodeling induced by AngII and sRAGE in an AngII-induced LV hypertrophy mice model.

## Introduction

Left ventricular hypertrophy (LV hypertrophy, LVH) is an important marker of organ damage in hypertension and also an independent risk factor for sudden death^1^, ventricular arrhythmias^2^, myocardial ischemia^3^, cardiovascular disease (CVD)^4^, and congestive heart failure^5^. Studies have shown that renin-angiotensin system (RAS), especially angiotensin II (AngII), exhibits important effects on development of cardiac hypertrophy and regulation of LV function^6^. Direct cardiac actions of AngII include stimulation of cardiac contractility; acceleration of protein synthesis, which results in cardiac hypertrophy; and activation of a membrane phospholipase^7^. Activation of RAS signaling is also intimately related with the expression level of receptor for advanced glycation endproducts (receptor for AGE, RAGE), which belongs to the immunoglobulin superfamily of cell surface molecules^8,9^. RAGE is a pattern-recognizing receptor that engages with other non-glycated peptides which commonly have multiple β-sheets, including high-mobility group box 1 protein (HMGB1)^10^, S100/calgranulin^11^, amyloid fibrils^12^, and Mac-1^13^. Since most of these ligands are common factors that are known to induce severe inflammatory diseases and as these factors can also activate RAGE-signaling pathway, RAGE has been purported to be a potential therapeutic target in various inflammatory diseases^14–16^. In our previous study, we demonstrated that RAGE has a pivotal regulatory role in AngII-induced cardiomyocyte hypertrophy through PKCs-EKR1/2 and NFκB-NLRP3-IL1β-signaling pathways *in vitro*, and demonstrated that soluble RAGE (sRAGE) as a decoy receptor for RAGE can attenuate AngII-induced cardiomyocyte hypertrophy *in vitro*^17^. However, the protective role of sRAGE in animal models of LVH has not been investigated.

In this study, we used 9.4T pre-clinical magnetic resonance imaging (MR imaging, MRI) instrument to obtain cardiac cinematic-MR (CINE-MR) images with high temporal and spatial resolution in live AngII-induced LV hypertrophy mouse models. CINE-MR imaging is very useful for the assessment of CVD, as it has high soft tissue contrast and spatial resolution and can also allow observation of biological functions, such as blood flow velocity and ratio, at live objectives^18,19^. However, since it is difficult to visualize the fast heartbeat of mice (300 beats/min) using MRI under 3.0T magnetic field, we used ultra-high field MRI to secure enough spatial and temporal resolution. The aim of this study was 1) to verify the cardiac functional and anatomical changes of AngII-induced LVH mouse model and 2) to determine the efficacy of treatment of sRAGE on attenuating AngII-induced LVH using CINE-MRI. For quantitative analysis, we obtained six parameters related to LV blood volume, as well as muscle volume and weight, from CINE-MR images. After *in vivo* MRI measurements, blood pressure, heart weight, and myocyte cross-sectional area (CSA) of cardiac specimen was obtained to confirm the findings on MRI.

## Materials and Methods

### LVH animal model

All procedures in this study were approved by the Institutional Animal Care and Use Committee of Yonsei University (approval reference number: 2014-0059) and were performed in accordance with the guidelines and regulation of the Association for Assessment and Accreditation of Laboratory Animal Care International. Male C57BL/6J mice at 8 weeks of age were purchased from the Jackson Laboratory (Bar Harbor, ME, USA) and used in this study. CINE-MRI was carried out before and after drug infusion in each group; saline (n = 10), AngII (n = 9), sRAGE (n = 10), and AngII + sRAGE (n = 10). To induce LVH, mice were anaesthetized by isoflurane (0.5%~1.0%/kg) and AngII (Sigma, St. Louis, USA) was infused into the mice subcutaneously at a concentration of 1.5 μg/min/kg for 4 weeks using osmotic mini-pumps (Alzet, model 2004, Cupertino, CA). sRAGE (Y-biologics, Daejon city, Korea, 2 μg/mouse) treatment was performed by daily intraperitoneal injection for 4 weeks. After CINE-MRI, mouse tail-cuff systemic blood pressure (BP) was measured on unanesthetized mice 4 weeks after infusion using BP-2000 Blood Pressure Analysis System (Visitech Systems, North Carolina, USA) according to the manufacturer’s instruction. After sacrifice, histological analysis for the extracted hearts was also performed, and body weight and tibia length were measured.

### *In vivo* MR imaging of LVH animal model

**Figure 1:**
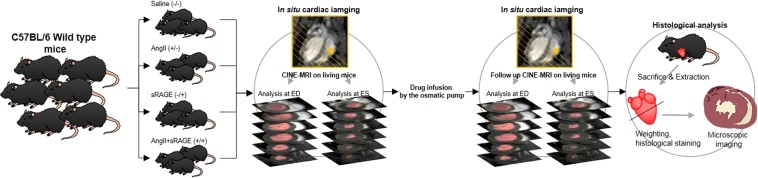
Schematics demonstrate the experimental process for the cardiac function tracking of angiotensin II-induced LVH models using *in situ* and 9.4T MRI.

*In vivo* cardiac CINE-MR imaging was conducted on a 9.4T animal MRI (BioSpec 94/20 USR, Bruker, Ettlingen, Germany) to confirm the difference of cardiac function among drug treatment groups (Fig. 1). It was acquired using a cylindrical volume RF coil (40 mm bore size) in C57BL/6 mice (n = 39) at 8 weeks of age before the implantation of osmotic pump. Anesthesia was induced by 3.0% isoflurane in oxygen and maintained using 1.0 ~ 2.0% isoflurane during MR imaging. Electrocardiogram and respiratory data were monitored during the scan. CINE-MR imaging was based on bright blood fast low angle shot (FLASH) CINE imaging: TR = 6 msec, TE = 2.1 msec, number of averages = 6, FOV = 32 × 32 mm^2^, acquisition matrix = 256 × 256, spatial resolution = 125 × 125 × 1,000 μm^3^, acquisition time = 270 sec, images in an acquisition = 35. To obtain the entire LV images of the heart, coronal view imaging of heart displaying cardiac apex and aortic arch was performed. Subsequently, CIEN-MR images were acquired in 1-mm thickness by short-axis slice, which was orthogonally oriented with the line joining from the end of cardiac apex and aortic arch, and we called this “short-axis slice”. After infusion of the drugs for 4 weeks, *in vivo* cardiac CINE-MRI was conducted again using the same method.

### Analysis of MR images

MR images were analyzed by ParaVision 5.1 software (Bruker, Germany). Cardiac function of LVH mice model was assessed by measuring of the left ventricle volume at ED and ES. In detail, the region of interest was drawn on LV, its area (A_LV_) was measured, and bright blood area of LV was separated by signal intensity. LV blood volume, myocardial volume and weight at the end of diastole (EDbv, EDmv, and EDmw), or end of systole (ESbv), were determined by the following formulas:

$${\rm{LV}}\,{\rm{blood}}\,{\rm{volume}}\,({\rm{mL}})=\sum _{n}{{\rm{A}}}_{{\rm{b}}n}{{\rm{t}}}_{n}$$

$${\rm{myocardial}}\,{\rm{muscle}}\,{\rm{volume}}\,({\rm{mL}})=\sum _{n}{({{\rm{A}}}_{{\rm{LV}}}-{{\rm{A}}}_{{\rm{b}}})}_{n}{{\rm{t}}}_{n}$$

$${\rm{myocardial}}\,{\rm{muscle}}\,{\rm{weight}}\,({\rm{g}})=\rho \sum _{n}{({{\rm{A}}}_{{\rm{LV}}}-{{\rm{A}}}_{{\rm{b}}})}_{n}{{\rm{t}}}_{n}$$

A_b_ is bright blood area of LV which is displayed as largest or smallest area from the acquired CINE-MR images, t is slice thickness, *n* is the number of short-axis slices, and *ρ* is myocardial density, assumed as 1.05 g/mL^20^. Ejection-fraction (EF) of heart was determined by:

$$\mathrm{EF}( \% )=\frac{{\rm{EDbv}}-{\rm{ESbv}}}{{\rm{EDbv}}}\times 100$$

The mean wall thickness of LV was calculated by ten lines that were drawn on LV wall of short-axis slice at ED.

### Histological analysis

After the *in vivo* MRI, all mice were sacrificed by perfusion after anesthesia, and their hearts were extracted. The hearts were weighted, fixed by 10% formalin solution over 24 h, and then cut transversely to generate paraffin block. For histological staining, 5-µm section was generated. After dewaxing and rehydration, wheat germ agglutinin (WGA) staining was conducted to analyze cardiomyocyte CSA using WGA-Alexa Fluor 594 (ThermoFisher Scientific, MA, USA)^21^. The stained tissue slides were randomly imaged at LV area of heart using fluorescence Microscope (Olympus, BX53, Tokyo, Japan), and estimated using Image J.

### Statistical analysis

All measured data were represented as mean and standard deviation from independent measurements. Statistical analyses were performed using Student’s t-test for verification of the changes contingent on drug administration in each group, and analysis of variance (ANOVA) with Bonferroni correction for comparison between experimental groups. The results were considered statistically significant when p < 0.05.

## Results

**Figure 2:**
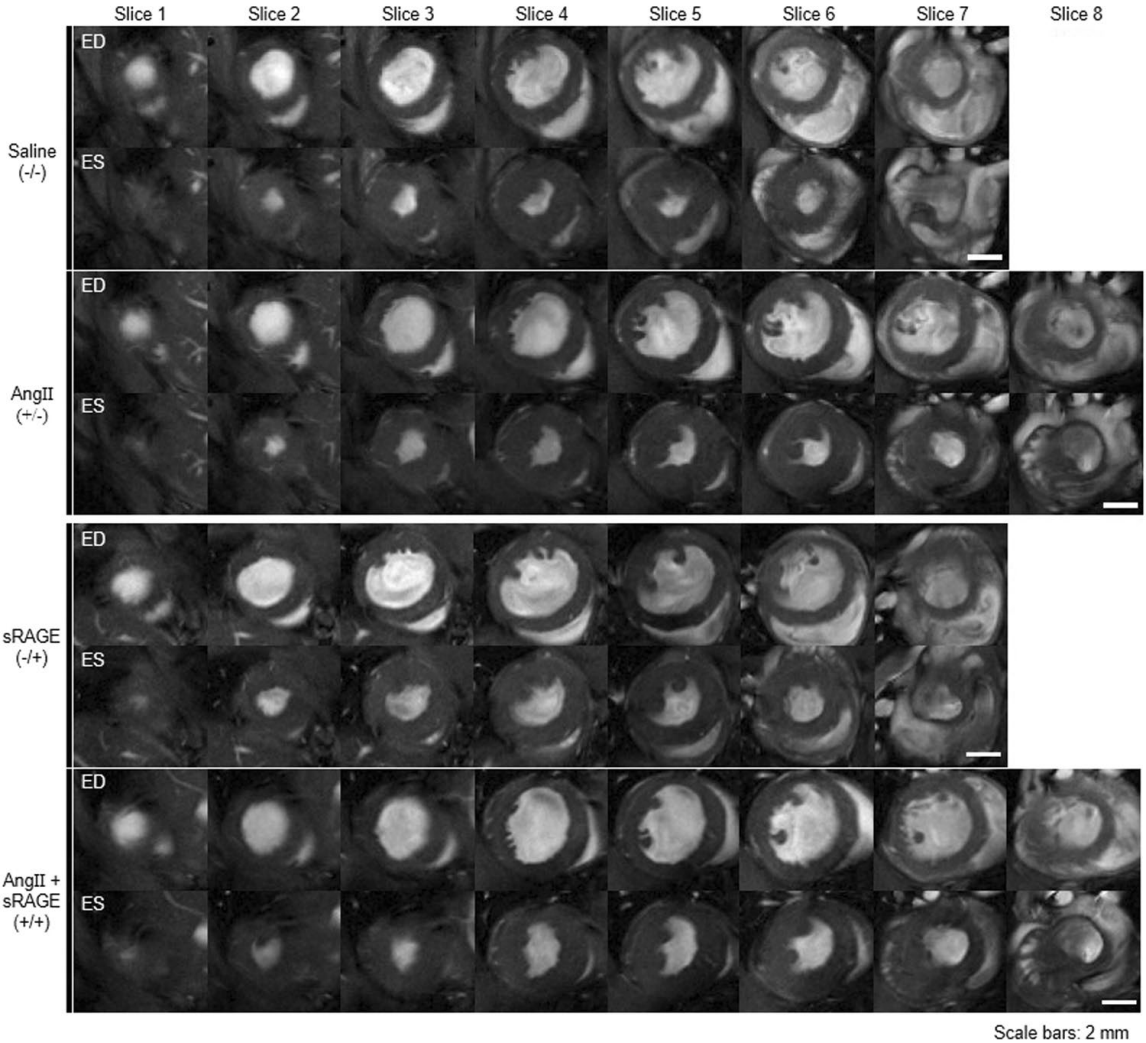
MR images displaying the heart of each experimental group at ED or ES after drug infusion by osmatic drug pump.

**Figure 3:**
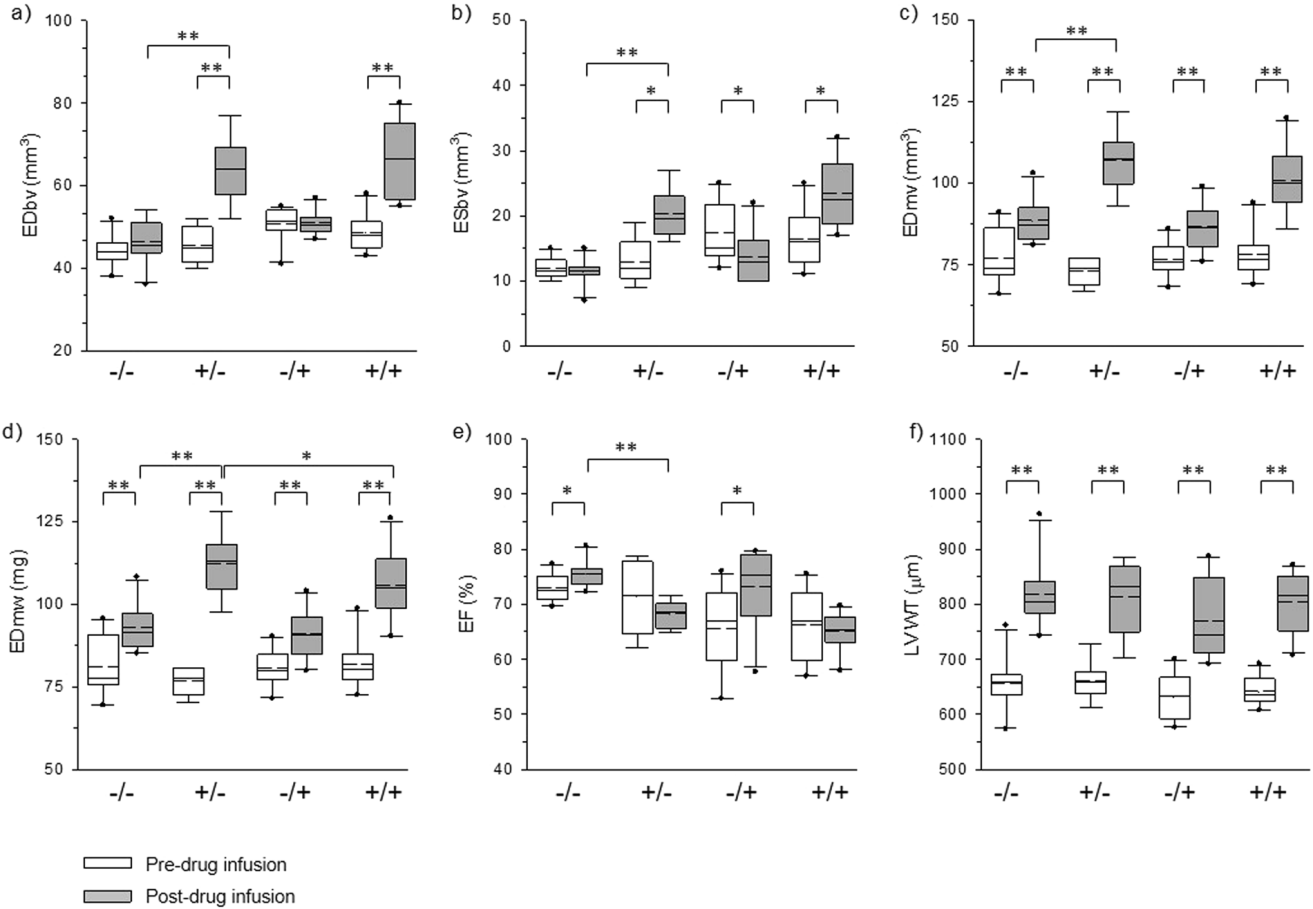
Graphs displaying the cardiac function and anatomy-indicating factors measuring from the cardiac CINE-MRI (**a**) EDbv, (**b**) ESbv, (**c**) EDmv, (**d**) EDmw, (**e**) EF of LV, (**f**) LVWT. −/−: saline group, +/−: AngII group, −/+: sRAGE group, +/+, AngII + sRAGE group. Straight line: median value, dashed line: mean value, ^*^p < 0.05, ^**^p < 0.01.

Figure 2 shows cardiac images of representative mouse in each group at the end of drug infusion. All images were obtained with bright blood FLASH CINE-MR images at ED or ES. The number of short-axis slices was not significantly different among experimental groups after infusion of drugs. The maximum difference of short-axis slice number was found in less than one slice. For a more precise analysis of MR images, six anatomical and functional parameters were selected as the principal variables indicating changes in cardiac function and morphology before and after implantation of osmotic drug infusion pump (Table 1). These parameters were as follows: EDbv, ESbv, EDmv, EDmw EF, and LV wall thickness (LVWT). Comparison of changes in these parameters between each treatment group after drug infusion are shown as box plots in Fig. 3. First, when parameters were determined before the implantation of drug pump, there was no statistically significant difference among groups (Fig. 3, white bars). All groups had healthy cardiac conditions, and also showed normal EF within the range of between 65% to 73%. The six parameters after drug infusion (gray bar) were compared to baseline (white bar) for each group. In both of AngII group and AngII + sRAGE group, statistically significant differences were found post-infusion for all parameters (p < 0.01) except EF. In contrast, saline group and sRAGE group showed same statistical trends in EDmw, EDmv, LVWT, and EF.

**Table 1:** The factors were selected as indicators of cardiac function in this study.

AngII/sRAGE		EDbv	ESbv	EDmv	EDmw	LVWT	EF
		(mm^3^)	(mm^3^)	(mm^3^)	(mg)	(μm)	(%)
Pre	−/−	44.0 ± 3.9	11.9 ± 1.7	77.1 ± 8.6	81.0 ± 9.0	656.4 ± 49.9	73.0 ± 2.6
	+/−	45.7 ± 4.1	13.4 ± 3.4	73.2 ± 3.8	76.9 ± 4.0	660.0 ± 35.0	71.5 ± 6.4
	−/+	50.7 ± 4.2	17.5 ± 4.6	76.8 ± 5.2	80.6 ± 5.5	632.2 ± 45.5	65.6 ± 7.8
	+/+	48.6 ± 4.4	16.5 ± 4.5	78.1 ± 7.4	82.0 ± 7.8	642.2 ± 27.4	67.4 ± 6.3
Post	−/−	46.6 ± 5.4	11.5 ± 2.0	88.6 ± 6.6	93.0 ± 7.0	818.4 ± 60.5	75.4 ± 2.4
	+/−	69.9 ± 19.5	26.3 ± 18.2	112.3 ± 17.7	118.0 ± 18.6	813.5 ± 66.2	68.3 ± 2.4
	−/+	51.0 ± 2.7	13.7 ± 3.9	86.8 ± 7.1	91.1 ± 7.5	769.7 ± 74.3	73.2 ± 7.2
	+/+	66.4 ± 9.5	23.4 ± 5.2	100.8 ± 10.5	105.8 ± 11.0	796.8 ± 53.0	65.4 ± 3.5

Next, we compared the six parameters after drug infusion between four experimental groups (Fig. 3, gray bars). AngII group showed significant increase in EDbv, ESbv, EDmv, and EDmw compared to saline group, demonstrating consistent induction of LVH. However, there was no significant difference in LVWT. For the assessment of inhibition effectiveness of sRAGE on AngII-induced LVH, we compared the six parameters after drug infusion between AngII and AngII + sRAGE groups. The results showed that EDmw was significantly lower in AngII + sRAGE group compared to AngII group (p < 0.05, Fig. 3d).

**Figure 4:**
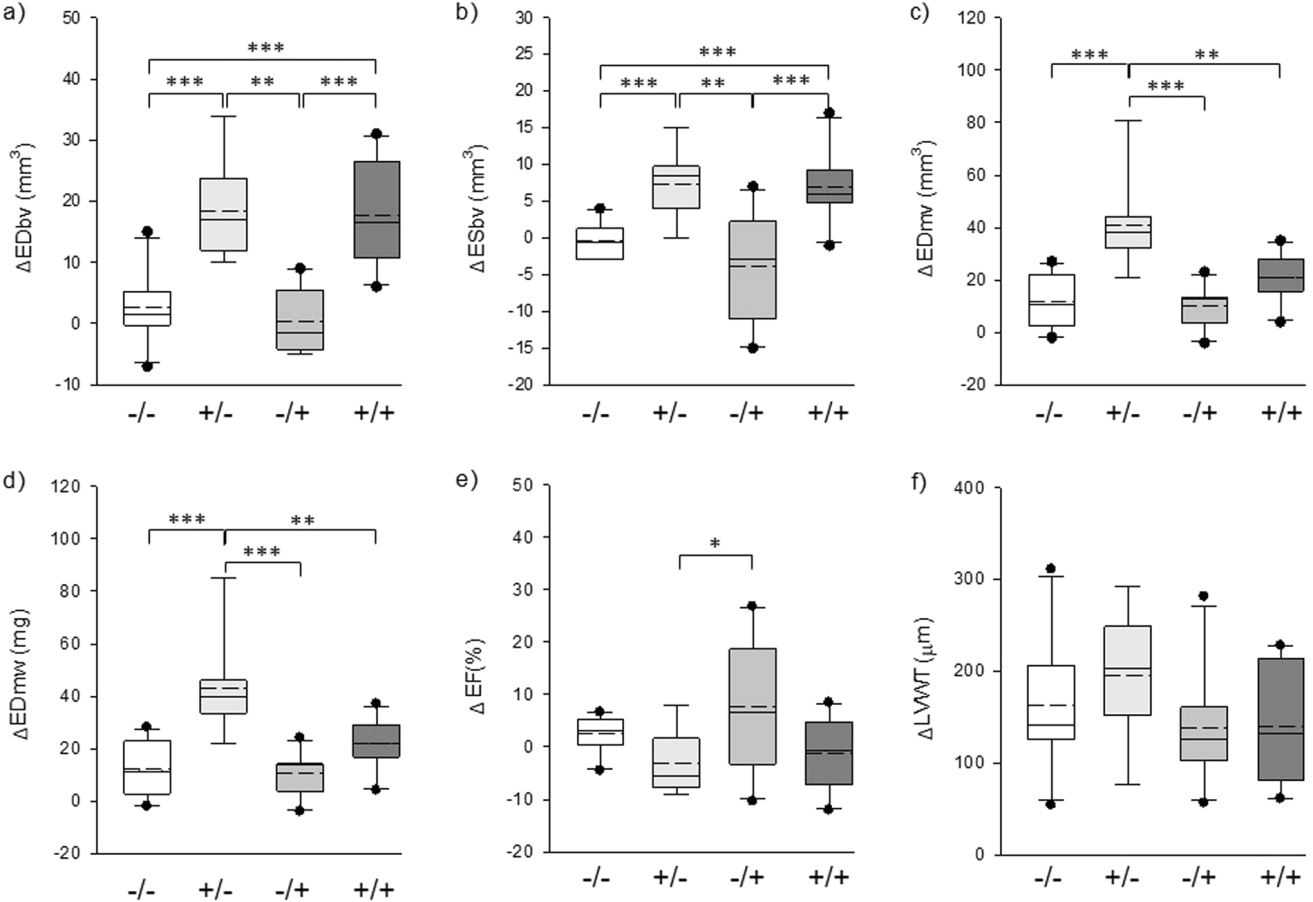
Graphs displaying the differential value of cardiac function and anatomy-indicating factors between before and after administration of AngII or sRAGE. (**a**) EDbv, (**b**) ESbv, (**c**) EDmv, (**d**) EDmw, (**e**) EF of LV, (**f**) LVWT. −/−: saline group, +/−: AngII group, −/+: sRAGE group, + /+, AngII + sRAGE group. Straight line: median value, dashed line: mean value, ^*^p < 0.05, ^**^p < 0.01, ^***^p < 0.0001.

When we compared delta values of the six parameters between each group by administration of AngII or sRAGE, the inhibitory effect of sRAGE on AngII-induced LVH was more obviously observed (Fig. 4). The results showed that AngII + sRAGE group had a significantly smaller increase in ΔEDmv and ΔEDmw compared to AngII group (Fig. 4c,d), while there was no significant difference for the changes in ΔLVWT. In contrast, no significant difference was observed between saline group and sRAGE group, suggesting that sRAGE did not have any anti-hypertrophy effect without concomitant stimulation with AngII.

**Figure 5:**
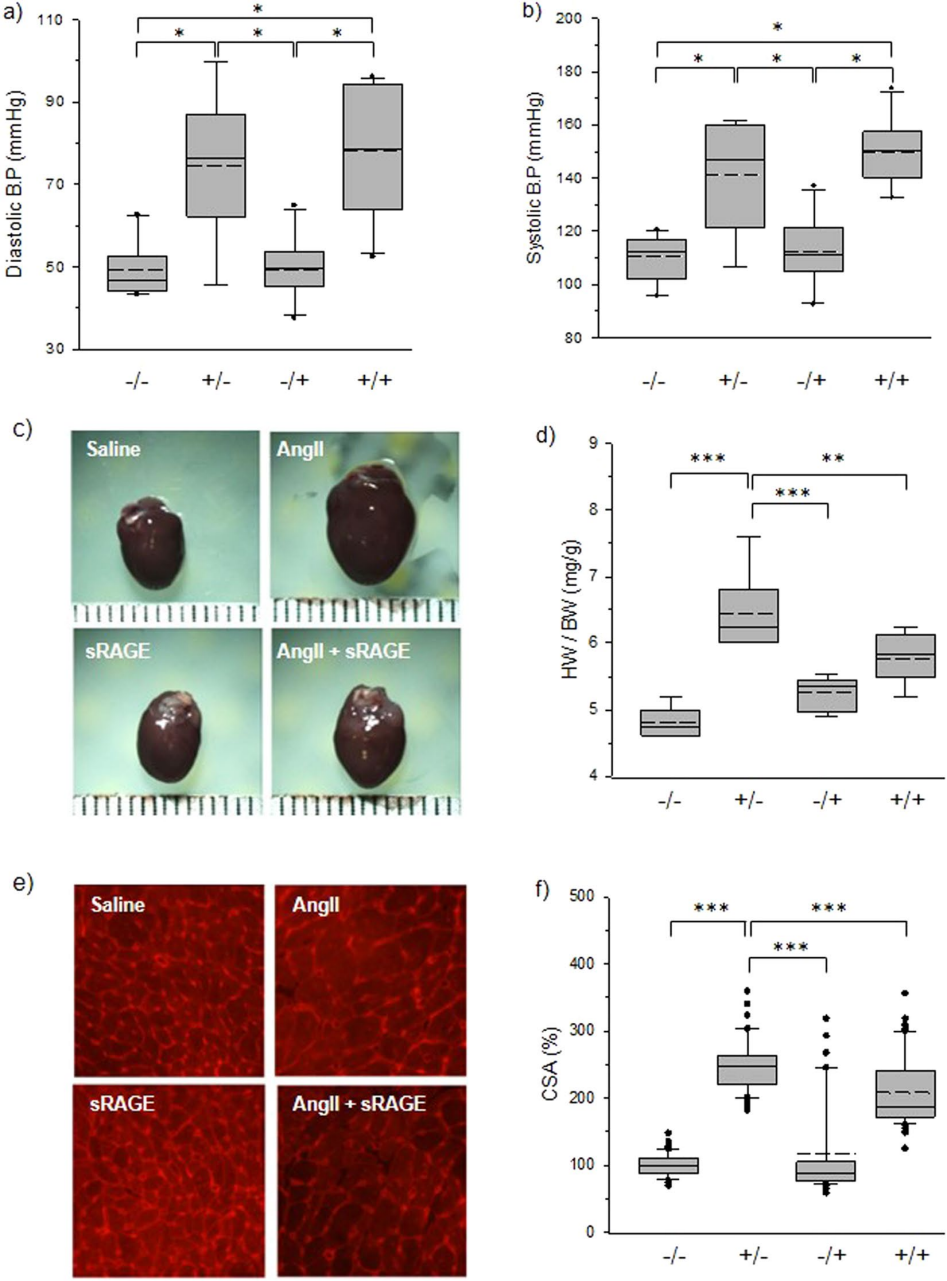
Blood pressure and histological analysis after CINE-MRI. (**a**) Diastolic blood pressure, (**b**) systolic blood pressure, (**c**) harvested heart images, (**d**) heart weight/body weight, (**e**) microscopic images of WGA-stained heart tissue, (**f**) CSA of myocardiocyte, ^*^p < 0.01, ^**^p < 0.05, ^***^p < 0.0001.

For confirmation of the above-mentioned results, blood pressure was also measured in all groups (Fig. 5a,b). There was significant increase in blood pressure in AngII group compared to saline and sRAGE groups, demonstrating the effectiveness of AngII osmotic pump infusion in elevating blood pressure. There was no significant difference in BP between AngII and AngII + sRAGE groups, demonstrating that the efficacy of sRAGE in attenuating LVH was BP independent. To verify the anatomical changes within the heart, all mice were sacrificed at the end of their second MRI examination.

The heart was extracted and weighed to determine the weight of the heart alone, and when controlled for body weight (Fig. 5c,d). LVH induced by AngII was evident through gross visualization of the whole heart (Fig. 5c). The heart weight/body weight in AngII group was significantly higher compared to that in AngII + sRAGE group (p < 0.05, Fig. 5d). WGA staining was conducted to analyze cardiomyocyte cross-sectional area (Fig. 5e). WGA-stained images were displayed to compare the size of cardiomyocytes between each group. Cardiomyocyte cross-sectional area was determined by estimating the area of myocytes in the region of interest, and normalized saline group was 100%. Cross-sectional area was greater in AngII group than in saline group mice (over 150%, p < 0.0001). Also, AngII group showed significantly greater mean CSA compared to AngII + sRAGE group (p < 0.0001), confirming the findings demonstrated by cardiac MRI. Expression levels of hypertrophic markers were also examined using heart tissues to support histological analysis (Fig. S1, see supporting information). β-MHC, ANP, and NFATc1 expression levels tended to increase with AngII-infused hearts, and sRAGE treatment tended to attenuate their expression levels.

**Figure 6:**
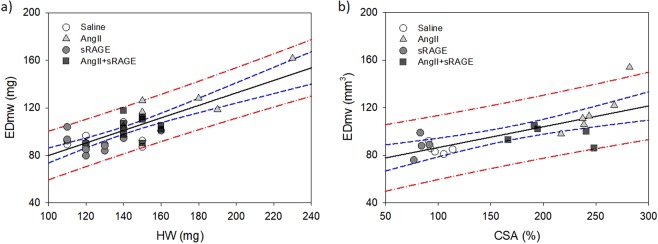
Linear regression analysis between CINE-MRI and histological data. (**a**) Linear regression between EDmw and HW (R^2^ = 0.63, p < 0.0001), (**b**) between EDmv and CSA (R^2^ = 0.54, p = 0.0002). Black straight line: Regression line, blue dashed line: 95% confidence band, red dot-dashed line: 95% prediction band.

To verify the correlation between CINE-MRI and histological data, linear regression analysis was conducted with n ≥ 4 samples from each group (Fig. 6). There was a good correlation between EDmw and HW (R = 0.79, R^2^ = 0.63, and p < 0.0001), with all data points being in the 95% prediction band (Fig. 6a). There was a good correlation between EDmv and CSA (R = 0.73, R^2^ = 0.54, and p = 0.0002) as well.

## Discussion

In the present study, we successfully detected AngII-induced functional and anatomical changes of hearts using *in vivo* real-time MR imaging method, and the acquired data indicated that sRAGE was effective in attenuating AngII-induced LVH. Previous studies have demonstrated AngII as an important mediator in the development of cardiac hypertrophy^22–24^, interstitial cardiac fibrosis^25,26^, and LV dysfunction^27–29^. Components of renin-angiotensin system, including AngII, constitute an autocrine/paracrine cardiac tissue renin-angiotensin system^30–33^. AngII receptors have been characterized in cardiomyocytes, cardiac fibroblasts^25,26,34^, and coronary arteries^26,35^. Although both angiotensin type 1 and 2 receptors (AT1R and AT2R) are present in cardiac and coronary tissue, many of the adverse effects of AngII on growth, myocardial fibrosis, and LV dysfunction are mediated through AT1R^24,34–37^. Accumulating evidence suggest that many of the adverse cardiac effects of AngII are triggered by reactive oxygen species (ROS), partially generated by an NADPH oxidase-dependent pathway^38–42^. These results can help with our understanding of the pathogenesis and biomolecular interactions involved in AngII and LVH.

In these previous studies of CVD using animal models, differences between experimental groups were confirmed by invasive and indirect methods such as blood examination or end-point assays, like tissue staining and western blotting^17,24,37,41,43,44^. The major disadvantage of these methods is that the results from one assay are limited and discontinuous. This impedes the study of biological or phenotypical changes during disease progression, since continuous observation is impossible in a living model.

To circumvent this problem using end-point assays, much effort has been exerted in developing non-invasive techniques such as MRI, computed tomography, ultrasound (US) imaging, and positron emission tomography. Among these, MRI is particularly useful for assessing CVD as it has high soft tissue contrast and spatial resolution, and can also allow observations of biological functions such as blood flow velocity and ratio^18,19,45,46^. Although echocardiogram is the most widely used imaging technique for experiments in small animal cardiovascular disease models, MRI is more suitable for monitoring of cardiac remodeling between baseline and post-intervention, due to its higher spatial resolution that allows better soft tissue contrast. Recently, US has also had significant improvement in spatial resolution, which enabled improved visualization of organs or tissues due to improvement of transducers and image-processing techniques^47,48^. Moreover, US can perform real-time imaging, allowing radiologist to observe lesions quickly and easily. However, since most US instruments adopt hand-held transducers, it is difficult to obtain accurate cross-sectional images in rapidly beating heart. Therefore, MRI is more suitable for follow-up studies that require a certain deep and cross-sectional image of heart for meta-image post-processing. Conventionally, LV volumetric analysis using US had applied a formula that was an estimation method from LV diameter at the parasternal short axis^49,50^. In our MRI study, about 300 heart image slices were analyzed from 39 mice, and we calculated the cardiac functional parameters by a method that was different from the conventional calculation method using one-slice image to get more accurate data. From this study, we found a good correlation between non-invasively obtained EDmw, EDmv with HW, and CSA, suggesting that 9.4T MRI is a useful tool for non-invasive monitoring and assessment of cardiac remodeling in mice models of LVH. One limitation of this study is that, since we did not perform echocardiography, we cannot confirm whether MRI is superior to echocardiography in assessing cardiac remodeling.

Generally, T1 mapping and late gadolinium enhancement imaging have been performed to detect myocardial fibrosis in CVDs and arrhythmia using MRI^51–53^. These imaging methods facilitate the visualization of myocardial extracellular volume, and several studies have verified that myocardial extracellular volume is moderately correlated with collagen volume fraction^54–57^. However, T1 mapping and late gadolinium enhancement imaging must inject a contrast agent intravenously, and they are suitable for CVD with moderate or severe fibrosis.

In this study, *in vivo* real-time 9.4T MR imaging was able to accurately measure the changes in various LV parameters such as EDbv, ESbv, EDmv, EDmw, EF, and LVWT in mice model of Ang II-induced LVH without contrast agent. Assessment of serial changes in LV function and overall remodeling time in a live animal is important for assessment of pathophysiology, as well as for testing efficacy of novel therapeutic agents. Therefore, real-time 9.4T MR imaging without contrast agents may be a useful tool for future cardiovascular research.

Moreover, real-time 9.4T MR imaging was also able to measure the AngII-induced LVH-attenuating effect of sRAGE for various LV parameters, with the comparison of delta values of the six parameters among groups (Fig. 4) being more obvious than that of the mean values (Fig. 3). This suggests that real-time 9.4T MR imaging may be a useful imaging tool for continuous assessment of the efficacy of treatment of therapeutic agents or drugs on LV function and remodeling in live animals.

Interestingly, statistically significant difference between AngII and AngII + sRAGE groups was only shown in myocardial-related factors without any changes in blood volume (Fig. 4c,d), with significant difference being demonstrated for EDmv and EDmw of AngII + sRAGE group compared to those of AngII group. This suggests that the efficacy of sRAGE in attenuating LVH induction by AngII was not dependent on changes in LV blood volume or blood pressure, but rather on the direct effect of sRAGE on cardiomyocyte hypertrophy. Although sRAGE showed LVH-attenuation effect, no reduction of blood pressure was detected after the treatment of sRAGE (Fig. 5a,b). Consistent with our results on blood pressure regulation of RAGE, Liliensiek *et al*. demonstrated that arterial blood pressure was not affected by endothelial specific RAGE deletion or sRAGE treatment^58^. However, our previous study showed the anti-hypertrophic effect of sRAGE through inhibiting inflammation-mediated signaling pathways, such as ROS and NFκB in cardiomyocytes^17^. To support our results, blockade of ROS and NFκB-mediated inflammation has been implicated in cardiac pathogenesis^59,60^. In addition, the association between inflammatory markers, left ventricular hypertrophy, and diastolic dysfunction was also suggested in human study^61^.

A key finding from this study was that 1) we were able to accurately demonstrate AngII-induced LVH by 9.4T MR imaging after 4-week administration of AngII, which was confirmed by gross histologic analysis. This result demonstrated that real-time 9.4T MR imaging may be useful for serial monitoring of changes in various parameters of LV function and remodeling such as EDbv, ESbv, EDmv, EDmw, EF, and LVWT in live animals; and that 2) the protective effect of sRAGE in attenuating AngII-induced LVH was demonstrated by 9.4T MR imaging and confirmed by gross histologic analysis, as well with their high correlativity. Since the efficacy of experimental drugs require assessment of changes in cardiac parameters from baseline to after intervention, accurate assessment is limited by histologic assessment. The results from our study demonstrate that 9.4T MR imaging may be a useful tool to assess the efficacy of various experimental drugs on changes in cardiac function and remodeling between baseline and post intervention.

## Conclusions

Cardiac function tracking using *in vivo* and real-time 9.4T MR imaging detected the cardiac remodeling induced by AngII, as well as the protective effect of sRAGE in attenuating AngII-induced LVH. Six parameters reflecting cardiac function and anatomy were successfully measured, and showed statistically significant differences between AngII and AngII + sRAGE groups. AngII infusion for 4 weeks was sufficient to induce anatomical changes of the heart, although the magnitude of changes in LVWT and EF did not differ significantly between those seen in saline group and AngII + sRAGE group. When we compared AngII and AngII + sRAGE groups, decrease in ΔEDmv and ΔEDmw was observed by the administration of sRAGE with statistical significance. The results from our study demonstrate that 9.4T MR imaging may be a useful tool to non-invasively assess the efficacy of various interventions on changes in cardiac function and remodeling between baseline and post-intervention.

## Supplementary information

Supplementary information
