# Supplementary material for: Radiological assessment of effectiveness of soluble RAGE in attenuating Angiotensin II-induced LVH mouse model using in vivo 9.4T MRI
Source: Sci Rep. 2019 Jun 11;9:8475. doi: 10.1038/s41598-019-44933-6 (PMC6559980; doi:10.1038/s41598-019-44933-6)
Supplement: Supplementary file 1 — Supplementary information [file 41598_2019_44933_MOESM1_ESM.docx]

**Supporting information:**

**Radiological assessment of effectiveness of soluble RAGE in attenuating Angiotensin II-induced LVH mouse model using *in vivo* 9.4T MRI**

*Dan Heo, PhD,^1^**^†^ Soyeon Lim, PhD,^2†^ Jiye Lee, BSc,^3^ Myung Eun Lee, PhD,^3^ Soyoung Cho, BSc,^3,4^ Jisu Jeong, MSc,^3,4^ Miran Seo, PhD,^3^ Sungha Park, MD, PhD,^3,5,6,7*^ Jaemoon Yang, PhD^1*^*

^1^Department of Radiology, Yonsei University College of Medicine, Seoul, Republic of Korea

^2^Institute for Bio-Medical Convergence, College of Medicine, Catholic Kwandong University,

Gangneung, Gangwon-do, Republic of Korea

^3^Severance Integrative Research Institute for Cerebral & Cardiovascular Diseases, Yonsei University College of Medicine, Seoul, Republic of Korea

^4^Graduate Program in Science for Aging & Yonsei Research Institute of Aging Science, Yonsei University, Seoul, Republic of Korea

^5^Cardiovascular Research Institute, Yonsei University College of Medicine, Seoul, Republic of Korea

^6^Division of Cardiology, Yonsei University College of Medicine, Seoul, Republic of Korea

^7^Brain Korea 21 Project for Medical Science, Yonsei University, Seoul, Republic of Korea

^†^ These authors contributed equally to this work.

*Co-corresponding author:

Jaemoon Yang, e-mail: [177hum@yuhs.ac](mailto:177hum@yuhs.ac) / T. +82-2-2228-0845 / F. +82-2-2227-8129

Sungha Park, e-mail: [SHPARK0530@yuhs.ac](mailto:SHPARK0530@yuhs.ac) / T. +82-2-2228-8455 / F. +82-2-2227-8129

***Reverse transcription polymerase chain reaction (RT-PCR)***

Heart tissues were freshly isolated from 2 weeks followed by infusion of Ang II. Total RNA from hearts was isolated using Qiazol® (Qiagen, Venlo, The Netherlands), according to the manufacturer's instructions. cDNA was synthesized using reverse transcriptase (Bio-Rad, California, USA) and equal amounts of cDNA were amplified using PCR reaction kit (Bioneer, Daejeon, Korea). The primer sequences are: ANP (sense 5’- CTG CTA GAC CAC CTG GAG GA-3’ and antisense 5’- AAG CTG TTG CAG CCT AGT CC -3’); α-MHC (sense 5’- CAT TCC CAA CGA GCG AAA GG-3’ and antisense 5’- GGG CCT GGA TTC TGG TGA TG-3’); β-MHC (sense 5’- TCG ATT TGG GAA ATT CAT CC-3’and antisense 5’- CGC ATA ATC GTA GGG GTT GT-3’); NFATc1 (sense 5’- CTG GGA GAT GGA AGC AAA GAC-3’ and antisense 5’- GCG ACT TGG TCT TGT GAA TAG GG -3’) and β-actin (sense 5’- CCA TGT ACG TAG CCA TCC AGG -3’ and antisense 5’- GAC AGT GAG GCC AGG ATA GAG C-3’). Each gene expression was normalized to β-actin. PCR products were separated on 1.5% agarose gels and stained with ethidium bromide.


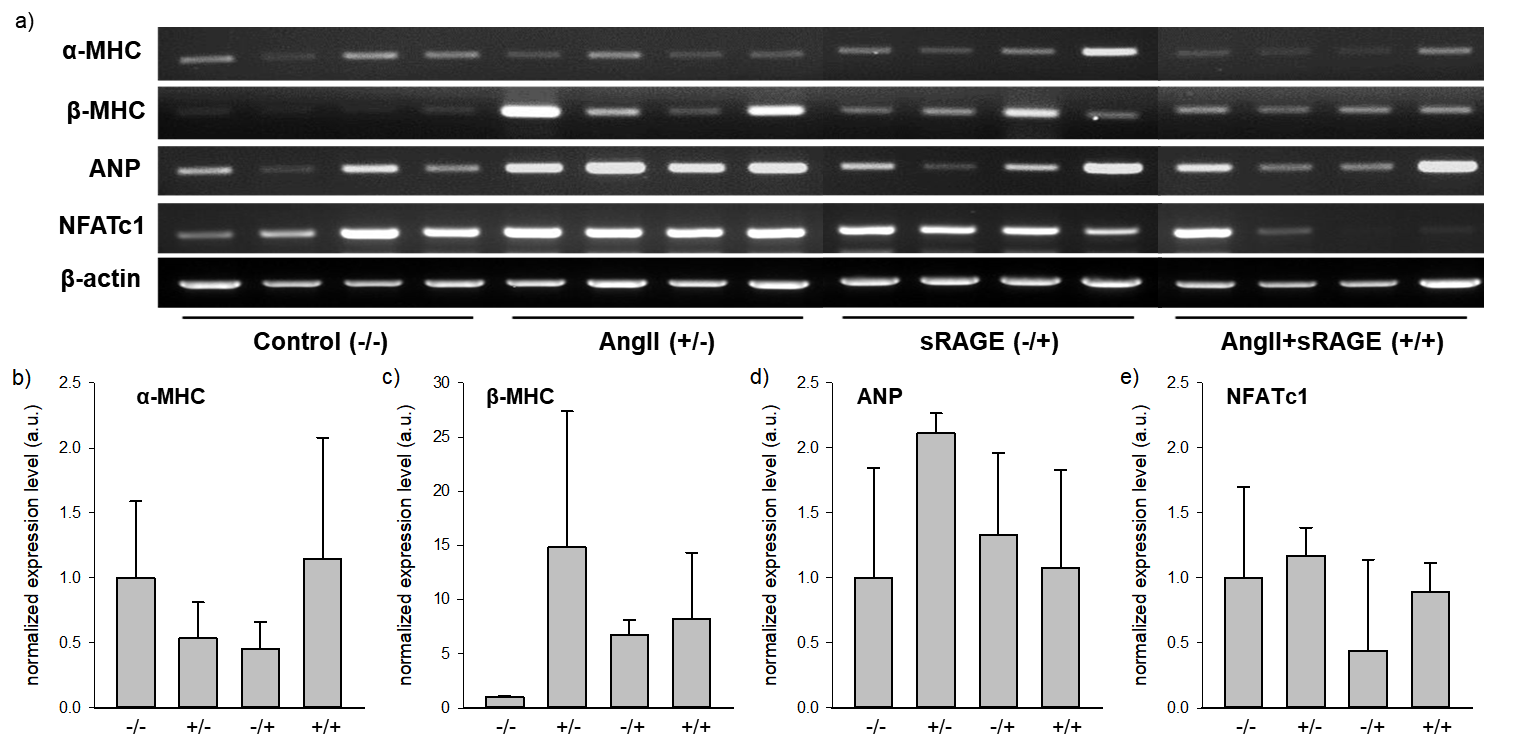


**Figure S1**. Expression levels of LVH-related makers obtained by RT-PCR. (a) Fluorescence images of the markers obtained from gel imager. (b) Expression level of α-MHC, (c) β-MHC, (d) ANP, (d) NFATc1. All expression levels were normalized by it of β-actin. This gel image cropped from different gels, Origianl gel images is displayed in figure S2.


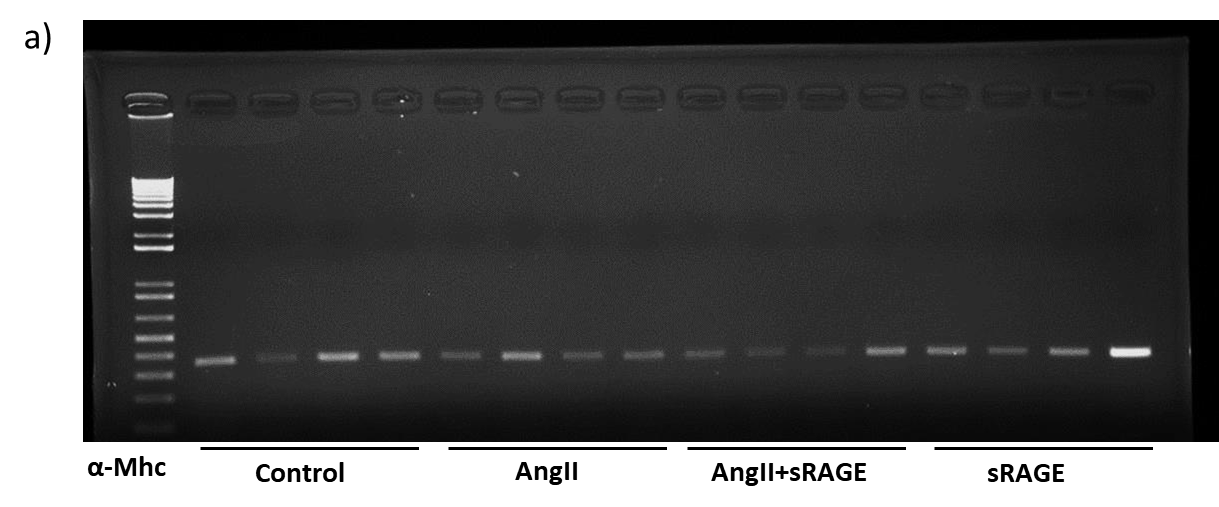

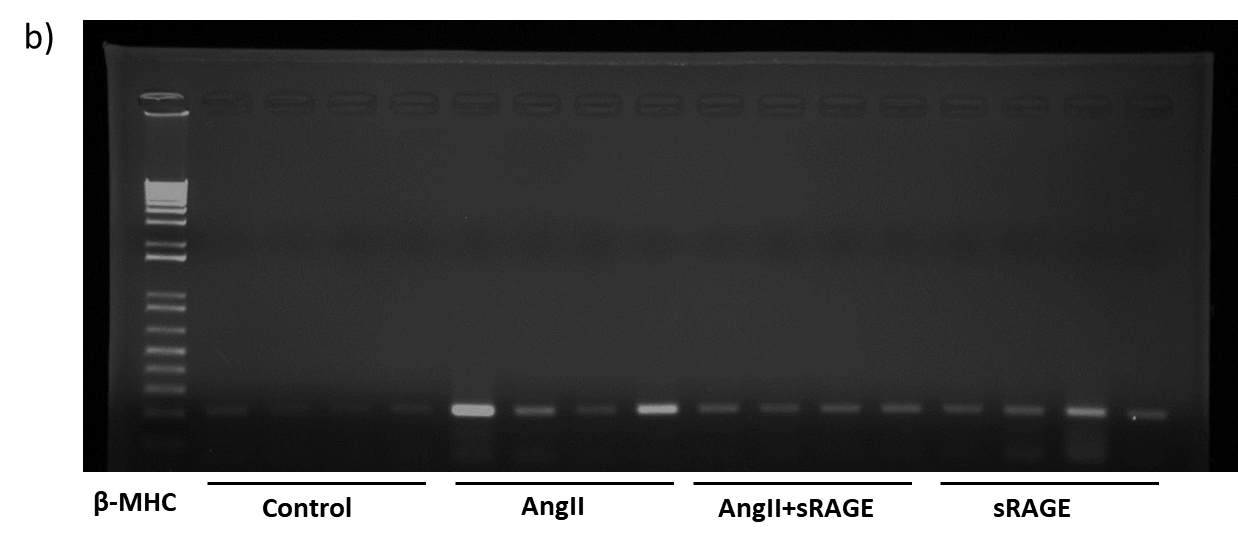


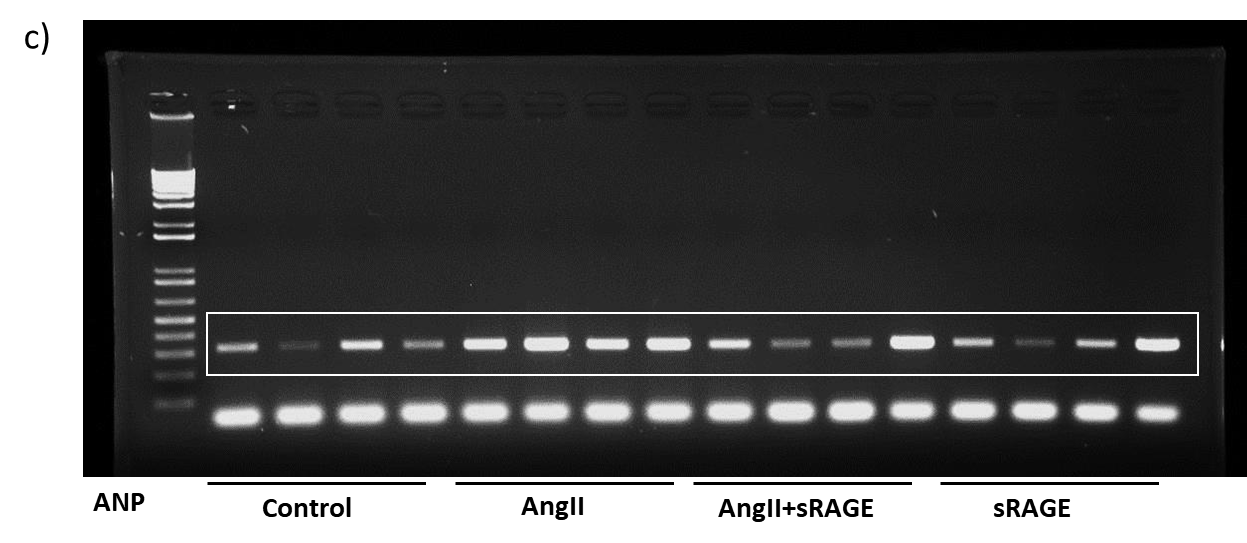


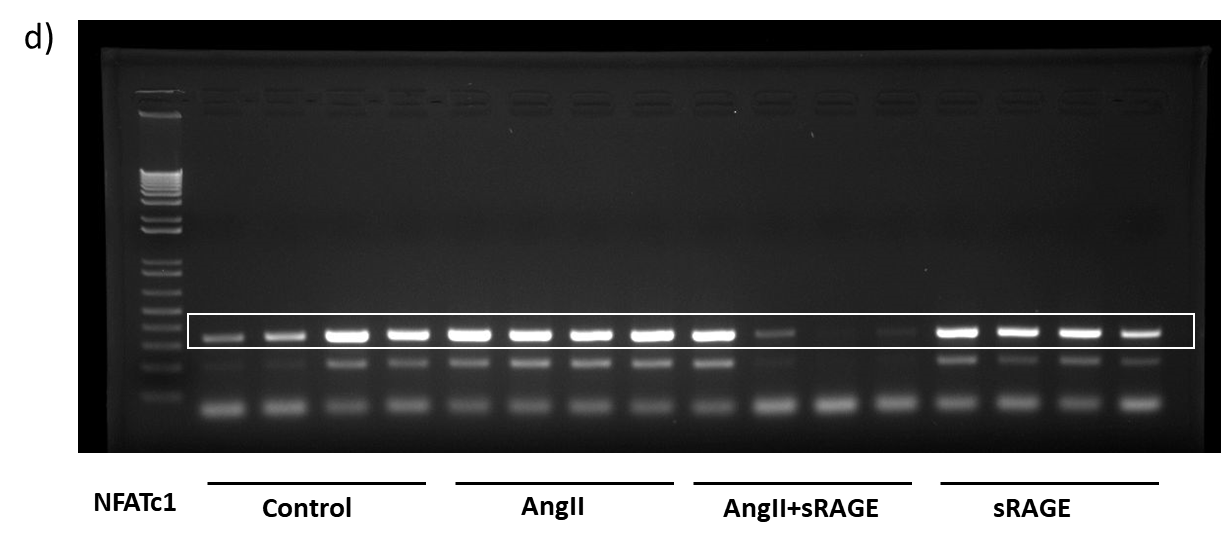


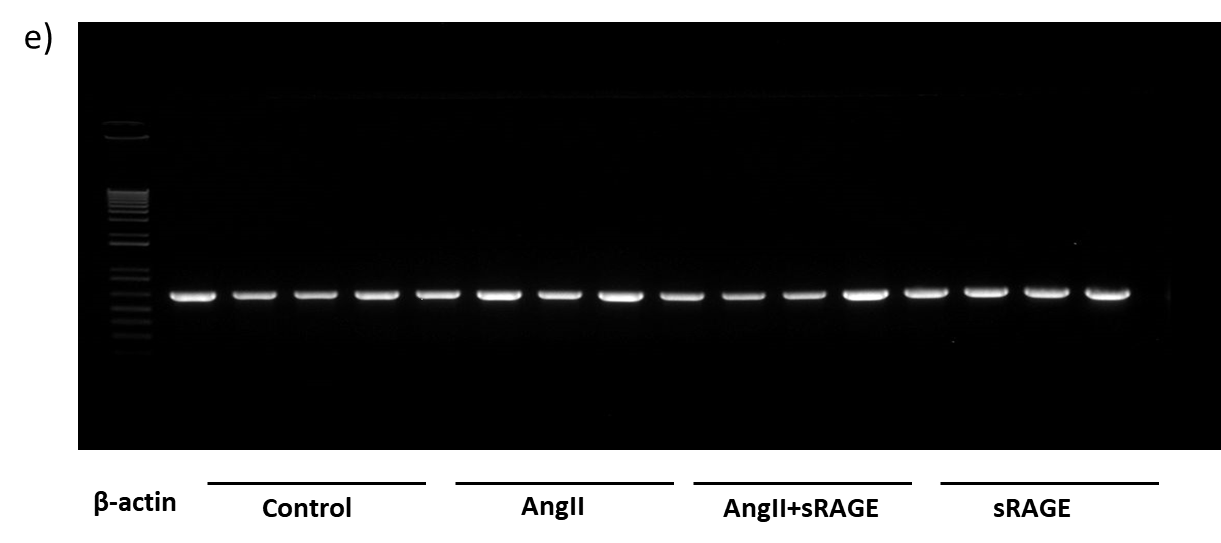


**Figure S2.** The original gel images of cropped images displayed in figure S1. (a) α-MHC, (b) β-MHC, (c) ANP, (d) NFATc1, and (e) β-actin.
